# Enabling Automated Device Size Selection for Transcatheter Aortic Valve Implantation

**DOI:** 10.1155/2019/3591314

**Published:** 2019-11-03

**Authors:** Patricio Astudillo, Peter Mortier, Johan Bosmans, Ole De Backer, Peter de Jaegere, Matthieu De Beule, Joni Dambre

**Affiliations:** ^1^FEops, Technologiepark-Zwijnaarde 122, Ghent, Belgium; ^2^Department of Electronics and Information Systems, UGent-imec, Technologiepark-Zwijnaarde 126, Ghent, Belgium; ^3^University Hospital Antwerp (UZA), Antwerp, Belgium; ^4^Department of Cardiology, Rigshospitalet University Hospital, Copenhagen, Denmark; ^5^Department of Cardiology, Erasmus MC, Rotterdam, Netherlands

## Abstract

The number of transcatheter aortic valve implantation (TAVI) procedures is expected to increase significantly in the coming years. Improving efficiency will become essential for experienced operators performing large TAVI volumes, while new operators will require training and may benefit from accurate support. In this work, we present a fast deep learning method that can predict aortic annulus perimeter and area automatically from aortic annular plane images. We propose a method combining two deep convolutional neural networks followed by a postprocessing step. The models were trained with 355 patients using modern deep learning techniques, and the method was evaluated on another 118 patients. The method was validated against an interoperator variability study of the same 118 patients. The differences between the manually obtained aortic annulus measurements and the automatic predictions were similar to the differences between two independent observers (paired diff. of 3.3 ± 16.8 mm^2^ vs. 1.3 ± 21.1 mm^2^ for the area and a paired diff. of 0.6 ± 1.7 mm vs. 0.2 ± 2.5 mm for the perimeter). The area and perimeter were used to retrieve the suggested prosthesis sizes for the Edwards Sapien 3 and the Medtronic Evolut device retrospectively. The automatically obtained device size selections accorded well with the device sizes selected by operator 1. The total analysis time from aortic annular plane to prosthesis size was below one second. This study showed that automated TAVI device size selection using the proposed method is fast, accurate, and reproducible. Comparison with the interobserver variability has shown the reliability of the strategy, and embedding this tool based on deep learning in the preoperative planning routine has the potential to increase the efficiency while ensuring accuracy.

## 1. Introduction

Transcatheter aortic valve implantation (TAVI) has become the preferred treatment for patients with aortic stenosis at high risk for surgical aortic valve replacement (SAVR) [1]. Recently, it was concluded that, for intermediate-risk patients, TAVI was similar to SAVR with respect to the primary end-point of death or disabling stroke [2, 3]. Very recent clinical data even show that TAVI is at least as good as SAVR in low-risk patients [4, 5].

The number of TAVI procedures is increasing each year rapidly [6], and considering the recent clinical data for low-risk patients will lead to an accelerated expansion in the coming years. As a result, scalability of the complete procedure, including preoperative planning, becomes an important aspect. Experienced operators can enlarge their volume of TAVI cases, for example, by increasing procedural efficiency. On the other hand, many new operators will need to be trained, which logically leads to increased risks due to their limited experience. When focusing on the preoperative planning, accurate automated detection of the aortic annulus dimensions directly from multidetector computed tomography (MDCT) images could not only increase efficiency but also at the same time reduce operator variability, thereby minimizing the impact of experience on TAVI sizing.

In this work, we present a deep learning method that can predict the aortic annulus perimeter and area automatically. The method is validated against an interoperator variability study to assess its accuracy. As a final step, the impact of the proposed method on the prosthesis size selection for both the Edwards Lifesciences and Medtronic transcatheter aortic bioprostheses was evaluated.

## 2. Materials and Methods

### 2.1. MDCT Imaging

This retrospective study used the anonymized data of 473 patients collected from multiple centra. The mean age of this cohort was 80.82 ± 7.18 years, and 55% of the patients were female. There were 36 bicuspid patients in this cohort. The patient data consisted of volumetric MDCT images which were acquired to plan a TAVI procedure. Therefore, all MDCT images were contrast-enhanced and contained a certain degree of aortic stenosis. The average row, column, and slice thickness of the MDCT images were 512.05 mm, 511.85 mm, and 0.83 mm. The aortic annular planes (AAP) were manually identified from the volumetric MDCT images using the standard method [7] and were used as input for this study. For this retrospective study, formal consent is not required.

### 2.2. Manual Detection

The border of the aortic annulus was manually identified from the aortic annular planes by observer 1. The data of observer 1 were considered the ground truth in this study. Observer 2 repeated this for 118 randomly selected patients in order to assess the interoperator variability. Both observers applied the same manual method, which consists of visual detection of the aortic annulus within the AAP and annotating it using Mimics Innovation Suite 18 (Materialise, Leuven, Belgium).

### 2.3. Automatic Detection

This study aims at automating the manual segmentation and derives clinical patient-specific information as a postprocessing step. Preprocessing of the ground truth images and aortic annulus annotations were necessary in order to prepare the data for training the deep learning models.

**Figure 1:**
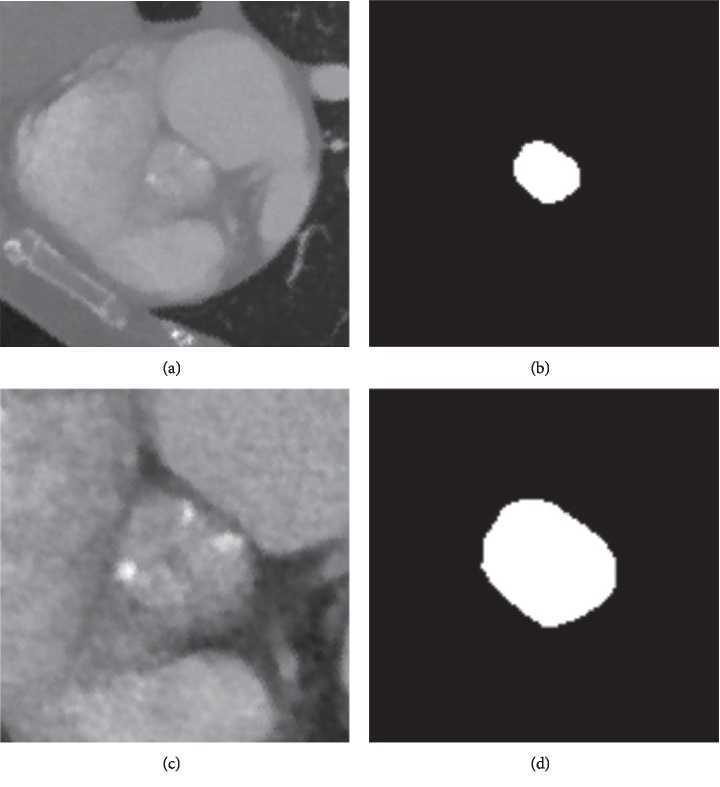
Examples of the aortic annular plane and the accompanying binary masks. The resampled and clipped aortic annular planes (a) and the binary masks (b) with different resolutions, 1.0 mm (c) and 0.5 mm (d).

The aortic annular planes were clipped and resampled in order to fit the neural networks' input. The aortic annular planes were resampled to an isotropic 1 mm resolution. As the deep learning network expected a 128 × 128 pixel plane as input, the resampled aortic annular planes were clipped around the center of the aortic annulus. A second isotropic 0.5 mm resolution was generated and clipped in the same manner in order to double the level of segmentation detail. Cubic spline interpolation was used in order to retain the original Hounsfield units in the resampled aortic annular planes (Figure 1). Binary masks were generated in order to teach the neural network how to segment the aortic annular plane using the ground truth annotations of the aortic annulus (Figure 1).

The deep learning model requires an architecture in order to process the resampled and clipped aortic annular planes and compare the output of the model with the binary masks. The used architecture was inspired by U-Net [8] and deep residual nets [9] and consisted of two paths: a downscaling and an upscaling path. The downscaling path extracted information from the aortic annular plane, and the upscaling path translated this information into a segmented aortic annulus. The final sigmoid activation function ensured that the output of the model contained probability values. The details of the deep learning architecture, training, and data-augmentation techniques are given in appendix A in Supplementary Materials ([Supplementary-material supplementary-material-1]).

The deep learning architecture was used during the training phase to teach a deep learning model to segment the aortic annulus from the aortic annular plane.

#### 2.3.1. Training

Two models were trained using the training dataset and validated with the validation dataset. One model was trained for each of the two resolutions (1 mm and 0.5 mm) of the aortic annular planes. The validation dataset consisted of the same 118 patients that were used for the interobserver variability study, and the training dataset consisted of the remaining 355 patients. The 36 bicuspid patients were distributed equally over the training and validation datasets.

### 2.4. Detection

After training one model for each resolution, a detection strategy was used to combine the output of both models and to derive patient-specific anatomical information: the area and perimeter of the aortic annulus.

**Figure 2:**
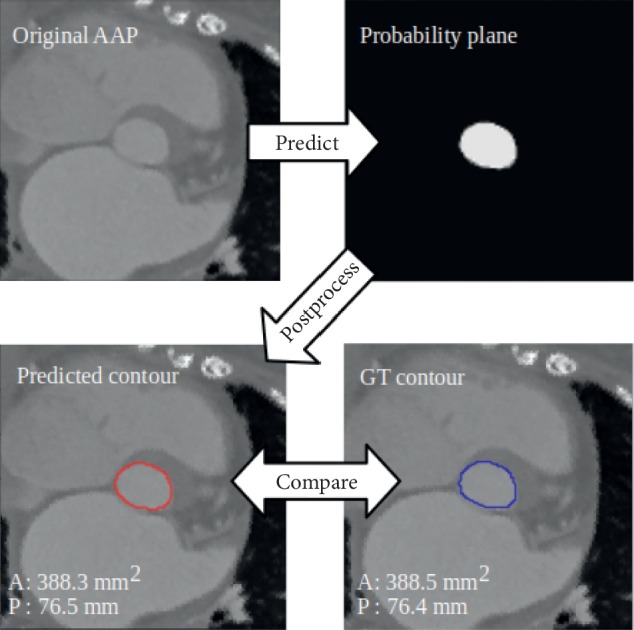
A general overview of the method: the model predicts the probability plane from the original aortic annular plane. The contours are detected, and the predicted area and perimeter are compared with the ground truth (GT).

The detection of the area and perimeter of the aortic annulus of a single patient was performed in two steps: a deep learning step and a postprocessing step. During the deep learning step, the aortic annular planes were analysed by both models, and the output was combined and normalized to a probability output that identified the region of interest. During the postprocessing step, the contour of the region of interest was located with canny edge detection [10] from the probability output. The area and perimeter were derived from this contour and serve as the final predicted output of the detection phase (Figure 2).

As a final step, the derived aortic annulus dimensions were used to assess correct prosthesis size. The perimeter was used to select the proper Medtronic Evolut TAVR prosthesis size (https://www.medtronic.com/content/dam/medtronic-com/products/cardiovascular/heart-valves-transcatheter/corevalve-evolut-r/documents/201709637EN-Evolut-PRO-TAV-in-SAV-Interactive-Sizing-Guide-FINAL.pdf), and the area was used to identify the Edwards Sapien 3 prosthesis size (https://www.accessdata.fda.gov/cdrh_docs/pdf14/P140031c.pdf) similar to the manufacturer's sizing matrix used in clinical practice.

### 2.5. Statistical Analysis

The Shapiro–Wilk test was performed to test for normal distribution, and none of the predicted distributions were normally distributed. Pearson correlation coefficient was computed to evaluate the correlation between model and both observers (with excellent correlation *R*^2^ > 0.9). The agreement between manual and the automatic landmark locations were evaluated using the nonparametric signed Wilcoxon test (with a significant *p* value <0.001). Bland–Altman analysis for area and perimeter between model and observer 1 and between both observers was performed.

### 2.6. Implementation

All the computational work was performed on a multicore computer with Titan *X* and P6000 GPUs (NVIDIA Corporation, Los Alamitos, CA). The models and the deep learning pipeline were developed with PyTorch v0.4.1 [11].

## 3. Results and Discussion

### 3.1. Results

The proposed method trained two models, and the detection phase was validated using the 118 patients used in the interoperator variability study. By using the same patients for validation and observer variability assessment, it was possible to compare the method with both observers.

The detection phase consisted of a deep learning phase and a postprocessing phase. The deep learning phase was validated by comparing the predicted segmentation (model) with the segmentation of both observers using the dice coefficient. The mean Dice score between model and observer 1 was 96% whereas the mean Dice score between both model and observer 2 and observer 1 and 2 was 89%. The higher mean Dice score between model and observer 1 is expected because the model was trained with the data from observer 1.

The postprocessing phase derived the area and perimeter from the predicted segmentation and was validated by comparing the predicted area and perimeter with the area and perimeter of both observers. When comparing the predicted anatomical measurements of the model with the data of both observers, there was no significant difference between the model and both observers for the area measurements. The mean paired difference for all measurements was around zero, which means that the predicted anatomical measurements could be used in the same manner as the output of observer 1 or 2 (Table 1).

**Figure 3:**
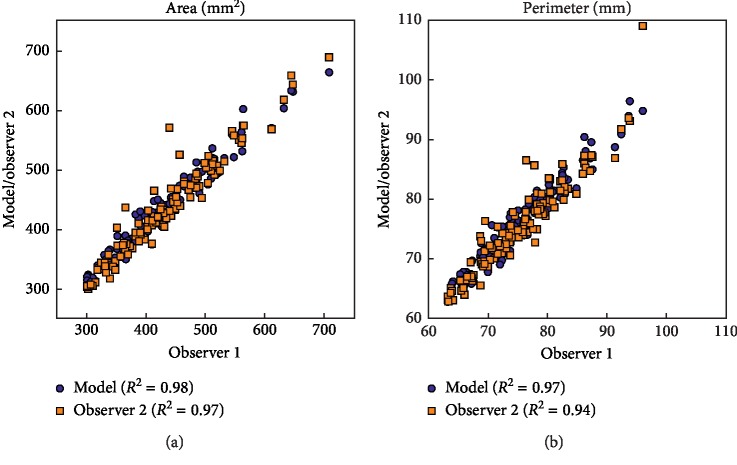
Scatter plots comparing the interobserver correlation for the area (a) and perimeter (b).

Excellent correction values were obtained between model and observer 1 for the area (0.98) and perimeter (0.97). The correlation values between observer 1 and 2 for the area (0.97) and perimeter (0.94) indicate that the manual method is accurate (Figure 3).

**Figure 4:**
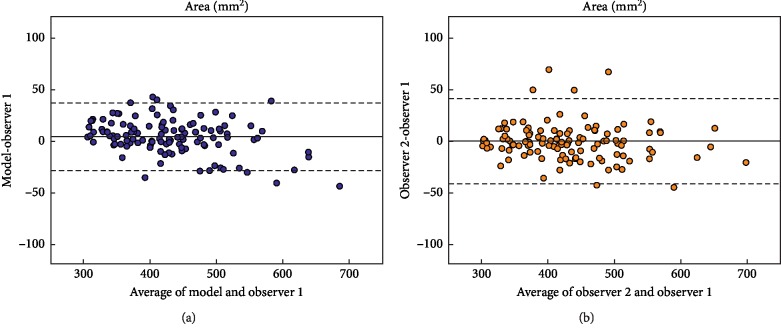
Bland–Altman plots comparing the aortic annulus area for model vs. observer 1 (a) and both observers (b).

**Figure 5:**
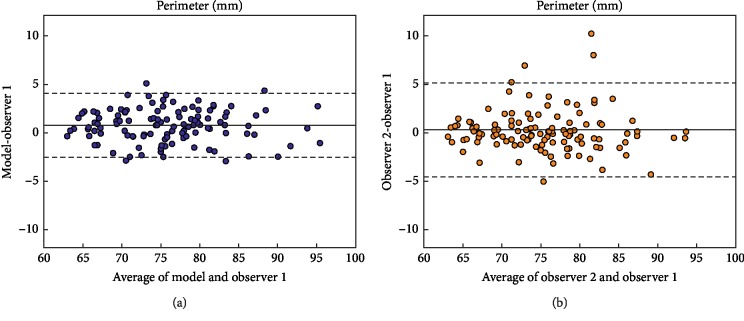
Bland–Altman plots comparing the aortic annulus perimeter for model vs. observer 1 (a) and both observers (b).

Bland–Altman plots of the predicted and measured (observer 1) area and perimeter are depicted in Figures 4 and 5. It is worth noting, when interpreting the Bland–Altman plots, that the model was repeatable since consecutive predictions per patient yielded the same output.

**Figure 6:**
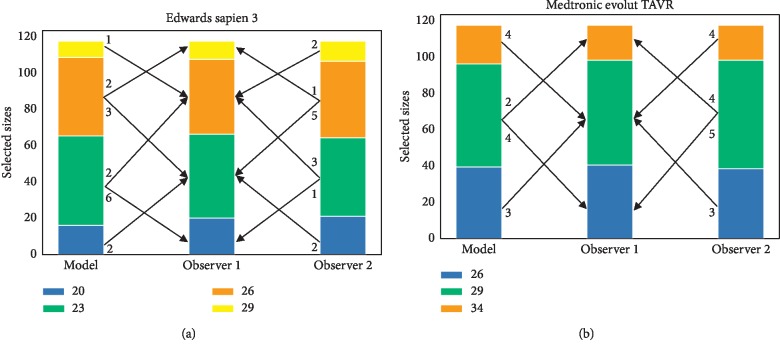
The agreement between prosthesis sizes from the Edwards Sapien 3 (a) and Medtronic Evolut TAVR sizing chart (b). The plots represent how many sizes were selected for each available device size based on the model, observer 1, and observer 2. The arrows between the plots indicate disagreement with observer 1 (under- or overestimation). The weights indicate the number of patients that were sized differently as compared to observer 1.

The validation of the segmentation abilities and the area and perimeter assessment were required to validate the method's ability to predict the correct prosthesis size (compared to both observers). The predicted area and perimeter were used to retrieve the Edwards Sapien 3 and Medtronic Evolut TAVR prosthesis sizes. The automatically selected valve sizes were compared with valve sizes resulting from the annular measurements of both observers. The ratio of agreement for Edwards Sapien 3 between model and both observers is almost equal: 0.86 between model and observer 1 and 0.88 between both observers. The ratio of agreement for the Medtronic Evolut TAVR prosthesis sizes between model and both observers is similar: 0.89 between model and observer 1 and 0.86 between both observers (Figure 6).

Finally, it is relevant to report the processing time of the manual and automated methods. The automatic processing time from aortic annular plane to segmentation, anatomical measurement, and prosthesis size is below 1 second.

### 3.2. Discussion

In this work, an automated method is proposed to facilitate and optimize the preoperative TAVI planning. It automatically predicts the area and perimeter of the aortic annulus based on MDCT images. The method has been validated on 118 patients to evaluate its accuracy, and the results show that the area and perimeter can be predicted in an automatic, reproducible, fast, and accurate way by combining the results of two networks followed by a postprocessing step. The differences between the manually obtained aortic annulus measurements and the automatic predictions are similar to the differences between two independent observers, which indicates a satisfying accuracy of the proposed approach. The area and perimeter have also been used to retrieve the suggested prosthesis sizes for the Edwards Sapien 3 and the Medtronic Evolute device. The automatically determined measurements result in device size selections that accord well with the device sizes selected by operator 1 based on his measurements, which again confirms the adequate model accuracy. The total analysis time from aortic annular plane to prothesis size is below 1 second.

In the literature, similar studies have been conducted. Queirós et al. proposed a method for detecting the correct TAVI prosthesis size from the aortic valve annulus area using aortic segmentation and statistical shape models [12]. Their full-automatic approach detected 92% of the prosthesis sizes and their semiautomatic approach 100%. This single-center study included 104 patients with a severe degree of calcification, mitral valve prosthesis, and pacemakers. The authors introduced an overlapping area of 35 mm^2^ and 40 mm^2^ between the 3 available prosthesis sizes of the Edwards Sapien 3 and XT. Unfortunately, this overlapping area makes it difficult to assess the true predictive power of the method and to compare with our results. Also, the final processing time was not reported in this study.

Our presented method is based on a different technique and goes, in our opinion, a step further than the work described in [12]. Our study includes both aortic valve annulus perimeter and area; therefore, the prosthesis size selection can be expanded to perimeter as well as area dependent devices. Next, multicenter data were used for training and validating the model, which may indicate robustness to unknown centers. No overlapping region was used in order to follow the manufacturers' guidelines and leave the final interpretation of the output of the method to the physician. Finally, the processing time is around one second per patient, which makes the method fast.

The method can detect the area and perimeter from the aortic annular plane within seconds, which may have an impact on reducing operator analysis time and errors in an exponentially growing market. If this method was combined with an automatic aortic annular plane detection method, the overall time reduction would be considerable. In addition to a time reduction of analysis and, thus, procedure planning, the physician saves time as he/she is liberated from this planning/analysis. Also, the analysis concerns an independent automated process that will enhance the output quality. Reduced overall TAVI costs may be obtained by embedding the method in software that allows manual corrections (e.g., to correct outliers). This embedding could also yield a continuous learning platform where the data of a new patient, validated by an expert, can be added to the training dataset, thus improving future detections.

Although the presented method has proven to be reliable, there are a few limitations related to the current approach. In a few cases, relatively large differences remain between the predicted area from our model and that from an individual human observer. Compared to observer 1, the largest overestimation of our model amounts to 10% and the largest underestimation to 9%. However, in those cases, observer 2 tended to agree with the predicted value (1% difference between observer 2 and the model). This may indicate that the model has generalized beyond the ground truth; in other words, it has learned to look beyond the few inaccuracies of its teacher. The maximum difference between the predicted perimeter and observer 1 was the same patient as the areas maximum difference (with a 7% overestimation). The minimum difference between predicated area and observer 1 was a 5% underestimation (a 3 mm difference).

It should be noted that the proposed method is not a TAVI planning tool, nor does it intend to replace the interventional cardiologist. There are other measurements required for the planning of a TAVI which are not included in this study. These measurements include the distance from the aortic annular plane to the ostium of the coronary arteries, the area of Sinus of Valsalva, sinotubular junction, and others and will be addressed in future work. It would also be interesting to measure the impact of this method prospectively.

## 4. Conclusions

In conclusion, this study shows that automated TAVI device size selection using the proposed method is fast, accurate, and reproducible. Comparison with the interobserver variability has shown the reliability of the strategy, and embedding this tool based on deep learning in the preoperative planning routine has the potential to increase the efficiency while ensuring accuracy.

## Figures and Tables

**Table 1:** A comparison of the anatomical measurements between model and both observers.

	Model vs. observer 1		Model vs. observer 2		Observer 1 vs. observer 2	
	Paired diff.	*p* value	Paired diff.	*p* value	Paired diff.	*p* value
Area (mm^2)^	3.3 ± 16.8	0.008	2.0 ± 22.4	0.046	1.3 ± 21.1	0.752
Perimeter (cm)	0.6 ± 1.7	0.0001	0.5 ± 2.6	0.0016	0.2 ± 2.5	0.513

Paired difference reported as mean ± standard deviation.

## Data Availability

The statistical data used to support the findings of this study are available from the corresponding author upon request. The anonymized image data used to support the findings of this study were supplied by FEops N.V. under license and so cannot be made freely available.

## Supplementary Materials

Supplementary Materials Figure 1: The overview of the deep learning architecture. Figure 2: The overview of the residual block: the input is expanded to the desired number of filters with a convolutional 2D layer with kernel size 1. After a sequence of convolutional layers with kernel size 3, batch normalization [3], and ReLU activation function [4], the output is summed with the output of the first convolutional layer followed by a final ReLU activation function. Table 1: Training details. All hyperparameters were obtained by performing k-fold cross-validation on the training set (with *k* = 5) and a fixed random seed. Table 2: Data-augmentation details. All parameters were similarly obtained as the training details.
