# Supplementary material for: Enabling Automated Device Size Selection for Transcatheter Aortic Valve Implantation
Source: J Interv Cardiol. 2019 Nov 3;2019:3591314. doi: 10.1155/2019/3591314 (PMC6875021; doi:10.1155/2019/3591314)
Supplement: Supplementary Materials — Figure 1: The overview of the deep learning architecture. Figure 2: The overview of the residual block: the input is expanded to the desired number of filters with a convolutional 2D layer with kernel size 1. After a sequence of convolutional layers with kernel size 3, batch normalization [3], and ReLU activation function [4], the output is summed with the output of the first convolutional layer followed by a final ReLU activation function. Table 1: Training details. All hyperparameters were obtained by performing k-fold cross-validation on the training set (with k = 5) and a fixed random seed. Table 2: Data-augmentation details. All parameters were similarly obtained as the training details. [file 3591314.f1.pdf]

Appendix A

Architecture

Overview of the architecture is depicted in Figure 1. The down- and up blocks define the down- and upscaling path of the architecture. The down block decreases the resolution of its input with a convolutional layer (with stride=2). The up block increases the resolution of its input with a transposed convolutional layer. The down- and up blocks contain a residual block followed by an alpha dropout layer (with p=0.5) [1] which maintains the self-normalising property within the model after performing a dropout. The residual block introduced an identity shortcut connection which enables deeper networks without degrading network performance due to poor convergence of learning [2].

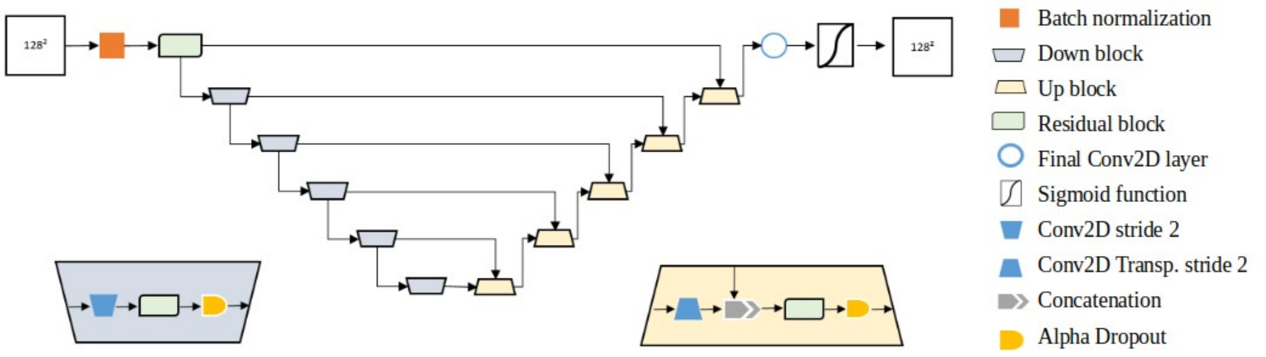

Figure 1: Overview of the deep learning architecture.

The residual block introduced an identity shortcut connection which enables deeper networks without degrading network performance due to poor convergence of learning [2] (Figure 2).

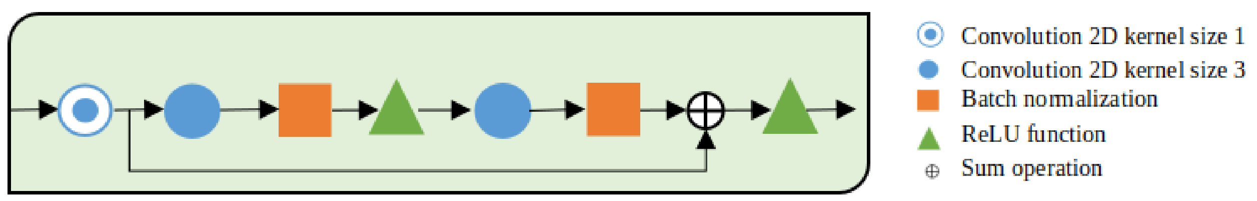

Figure 2: The overview of the residual block: the input is expanded to the desired number of filters with a convolutional 2D layer with kernel size 1. After a sequence of convolutional layers with kernel size 3, batch normalisation [3] and ReLU activation function [4], the output is summed with the output of the first convolutional layer followed by a final ReLU activation function.

Training

The Adam optimizer [5] was used to train each model with the generalized Dice loss [6] as an objective function for a maximum number of 10.000 epochs. The initial learning rate was 1e-2 but

was reduced after each epoch using the following formula: $1e\text{-}2/\sqrt{epoch}$. An L2 regularizer with a small lambda of 1e-3 was used to prevent overfitting. The weights of the convolutional layers were initialized with orthogonal random matrices [7] with a gain of $\sqrt{(2)}$ and biases were set to a small value of 0.1. All hyper-parameters were obtained by performing k-fold cross-validation on the training set (with k = 5) and a fixed random seed (Table 1).

**Optimizer**	Adam [5]	**Learning rate**	1e-2	**Weight init**	Orthogonal matrices [7]
**Objective**	Dice [6]	**Learning decay**	$1e\text{-}2/\sqrt{epoch}$	**Bias init**	0.1
**Max. epochs**	10000	**Regularizer**	L2 with λ=1e-3	**K-folding**	k=5

Table 1: Training details. All hyper-parameters were obtained by performing k-fold cross-validation on the training set (with k = 5) and a fixed random seed.

Data-augmentation

A high capacity model and data-augmentation handled the bias-variance trade-off specific to statistics and machine learning. The high capacity model yielded a low bias because it could identify relevant relations between features and target outputs. Data-augmentation lowered the high variance caused by the small size of the training set. The model was able to generalize well beyond the training set with a low bias and low variance.

The following data augmentation strategies were used: random rotation, random value dropout and random calcification placement, each with probability 0.5 of occurring during sample generation. Random rotation rotates the input images with a random angle (sampled between -45 and 45 degrees) to increase the robustness of the model against rotation. Random value dropout fills randomly positioned squares in the aortic annular planes with 0's, which is similar to the working of dropout [8] but was added to control the Hounsfield values of the input before the batch normalisation of the model. Most of the aortic annular planes contained an amount of calcification around the aortic annulus which interfered with the accuracy. Therefore the addition of random calcification in the aortic annular plane was used during training. This addition increased the robustness of the model for calcified areas. It was achieved by filling randomly positioned squares in the aortic annular planes with Hounsfield values that represent calcification. The calcification values were sampled from a normal distribution (mu = 1100 and sigma = 50, which were obtained from the training cohort) (Table 2).

**Rotation**	Rotates the input images with a random angle: sampled between -45 and 45 degrees

**Calcification placement**	Add random areas of calcification in the input images: samples from normal distribution μ=1100 and σ = 50.

Table 2: Data-augmentation details. All parameters were similarly obtained as the training details.

References

1. Klambauer G, Unterthiner T, Mayr A, Hochreiter S. Self-normalizing neural networks. InAdvances in neural information processing systems 2017 (pp. 971-980)
2. He K, Zhang X, Ren S, Sun J. Deep residual learning for image recognition. In Proceedings of the IEEE conference on computer vision and pattern recognition 2016 (pp. 770-778).
3. Ioffe S, Szegedy C. Batch normalization: Accelerating deep network training by reducing internal covariate shift. arXiv preprint arXiv:1502.03167. 2015 Feb 11.
4. Maas AL, Hannun AY, Ng AY. Rectifier nonlinearities improve neural network acoustic models. InProc. icml 2013 Jun 16 (Vol. 30, No. 1, p. 3).
5. Kingma DP, Ba J. Adam: A method for stochastic optimization. arXiv preprint arXiv:1412.6980. 2014 Dec 22.
6. Sudre, C.H., Li, W., Vercauteren, T., Ourselin, S. and Cardoso, M.J., 2017. Generalised dice overlap as a deep learning loss function for highly unbalanced segmentations. In *Deep learning in medical image analysis and multimodal learning for clinical decision support* (pp. 240-248). Springer, Cham.
7. Saxe AM, McClelland JL, Ganguli S. Exact solutions to the nonlinear dynamics of learning in deep linear neural networks. arXiv preprint arXiv:1312.6120. 2013 Dec 20.
8. Srivastava N, Hinton G, Krizhevsky A, Sutskever I, Salakhutdinov R. Dropout: a simple way to prevent neural networks from overfitting. The journal of machine learning research. 2014 Jan 1;15(1):1929-58.
